# Recent Advances in Anti-Cancer Drugs

**DOI:** 10.3390/ijms26178446

**Published:** 2025-08-30

**Authors:** Christiana M. Neophytou

**Affiliations:** Department of Life Sciences, European University Cyprus, Nicosia 2404, Cyprus; c.neophytou@euc.ac.cy

Despite the significant advancements in anti-cancer research in the last two decades, the development of anti-cancer therapeutics remains a central focus in oncology. The persistent burden of cancer and the complexity of tumor biology drive the need to improve current therapeutic regimens and the discovery of novel approaches. In recent years, the field has focused on the shift from conventional cytotoxic agents toward targeted therapies [1,2,3,4,5,6,7], compounds that can modulate the immune system [8,9,10,11,12], and treatments personalized to the genetic background of patients and ways to re-shape the microenvironmental landscape of tumors [13,14,15,16,17]. Despite this progress, critical gaps remain, particularly when it comes to overcoming drug resistance, designing efficient personalized therapy, and effectively targeting the tumor microenvironment (TME).

This Special Issue of IJMS comprises eight original contributions that highlight emerging trends, promising molecules, and novel biological targets across a spectrum of cancers. Several articles underscore the importance of precision medicine and the integration of molecular insights into therapeutic decision-making. For instance, the study on TP53 mutations and platinum resistance in high-grade serous ovarian cancer [18] offers compelling evidence in favor of incorporating genetic profiling into treatment planning, potentially improving therapeutic outcomes for resistant cases. Many studies have previously highlighted the role of TP53 [19,20,21], as well as other proteins such as BRCA1/2, PIK3CA, and KRAS, in resistance across multiple cancer types [22,23,24,25,26], indicating how integrating mutational profiles can refine therapeutic strategies, particularly when resistance to conventional chemotherapy emerges.

Two studies published in our Special Issue focus on the TME, which has been long recognized as a key player in therapeutic resistance and disease progression. One explores leukotriene inhibitors in chronic lymphocytic leukemia [27], suggesting new avenues for disrupting tumor–immune crosstalk. Another provides a comprehensive review of the TME’s role in pancreatic cancer immunotherapy [28], describing both current approaches and future potential strategies for enhancing immune responsiveness in immunosuppressive tumors. In recent years, manipulation of the TME has gained considerable attention as a strategy to enhance therapeutic efficacy, particularly in treatment-resistant tumors [29,30,31].

Innovation in the development of new pharmaceutics is also represented robustly. The synthesis and evaluation of oleanolic acid dimers [32] and silver complexes of antifungal agents [33] open up new possibilities for drug repurposing and structural modification to enhance anti-cancer activity. Drug repurposing and structural modification of existing compounds represent critical strategies in cancer therapy, offering accelerated approaches for treatment development by optimizing known pharmacological profiles and reducing the time and cost associated with novel drug discovery [34,35,36]. Similarly, the biodistribution and toxicity evaluation of [211At]PSMA-5 [37] in preclinical models marks an important step forward in alpha-particle radiotherapy for prostate cancer, a strategy that holds immense potential for targeting metastatic lesions with high specificity and lethality [38].

From a systems biology perspective, a bioinformatics-driven analysis of glycosylation-related genes in colorectal cancer [39] and a systematic review on monocarboxylate transporter 1 (MCT1) [40] offer valuable insights into metabolic vulnerabilities and transport pathways that could be exploited in therapy. These studies underscore the importance of integrative approaches—combining omics data, molecular profiling, and experimental validation—to uncover actionable targets [41,42].

Collectively, the papers in this Special Issue address notable gaps in current anti-cancer strategies: resistance to standard therapies, the elusive role of the TME, the need for innovative delivery systems, and the identification of underexplored molecular targets. They also reflect a broader movement in oncology research toward multi-dimensional, patient-tailored treatment approaches.

Future research should emphasize

- The development of reliable biomarkers to predict drug response;
- Combination approaches to overcome chemoresistance;
- Strategies for modifying the TME to enhance drug delivery and efficacy;
- Expansion of theranostic tools in nuclear medicine;
- Integration of artificial intelligence and multi-omics in drug discovery;
- Continued exploration of natural products and repurposed compounds.

I extend my sincere gratitude to the contributing authors for their high-quality work, and to the reviewers for their critical insights. Together, these contributions advance our understanding of cancer therapeutics and inspire continued innovation in the field.
